# Automated Polymer Purification Using Dialysis

**DOI:** 10.3390/polym12092095

**Published:** 2020-09-15

**Authors:** Timo Schuett, Julian Kimmig, Stefan Zechel, Ulrich S. Schubert

**Affiliations:** 1 Laboratory of Organic and Macromolecular Chemistry (IOMC), Friedrich Schiller University Jena, Humboldtstr. 10, 07743 Jena, Germany; timo.schuett@uni-jena.de (T.S.); julian.kimmig@uni-jena.de (J.K.); stefan.zechel@uni-jena.de (S.Z.); 2 Jena Center of Soft Matter (JCSM), Friedrich Schiller University Jena, Philosophenweg 7, 07743 Jena, Germany

**Keywords:** polymer purification, automation, dialysis, high-throughput experimentations

## Abstract

The automated dialysis of polymers in synthetic robots is described as a first approach for the purification of polymers using an automated protocol. For this purpose, a dialysis apparatus was installed within a synthesis robot. Therein, the polymer solution could be transferred automatically into the dialysis tube. Afterwards, a permanent running dialysis could be started, enabling the removal of residual monomer. Purification efficiency was studied using chromatography and NMR spectroscopy, showing that the automated dialysis requires less solvent and is faster compared to the classical manual approach.

## 1. Introduction

The ongoing digitalization has been significantly influencing chemical research over the last few years [1]. One major aspect in this field is the automation of chemical processes [2], and associated with that, the high-throughput synthesis of compounds [3,4]. For this purpose, flow-chemistry [5]/microfluidics [6,7,8,9] as well as robot-based synthesis can be performed, enabling a fast and more efficient screening of chemical reactions and processes. In particular, robotic systems feature the benefit of performing experiments simultaneously and thus, of performing more experiments in shorter timeframes [10]. Furthermore, the modern tools in such robotic systems enable nearly a complete investigation of all compounds such as liquids, solids, and gases. Additionally, reactions can be performed in multiple fashions by applying different reaction parameters, e.g., temperature, pressure.

Consequently, such systems were also utilized in polymer science [11] and, therefore, it was possible to elucidate polymer kinetics [12] and copolymerization parameters [12], as well as to accelerate reaction screening [13,14] or to perform the sequential addition of monomers [15]. Exemplarily, controlled radical polymerizations, such as reversible addition-fragmentation chain transfer polymerization, [16] were studied in detail using synthetic robots. Furthermore, a screening of polymers for a certain property, such as lower critical solution temperature (LCST), was possible using such an approach [17].

However, those systems were mainly optimized for the synthesis part of a reaction/polymerization. In some cases, the characterization is problematic due to the high number of samples due to the large outcome of the robot-based synthesis. On the other hand, the purification can rarely be performed using such robots. For polymers in particular, it is quite challenging to perform purification steps automatically since the properties of polymers, e.g., glass transition or melting temperature, solubility, and viscosity vary over a wide range. Therefore, high-throughput purification systems work by applying parallelization and manual experiments [18].

One of the first descriptions of an automated polymer purification method that was published used a short column in order to remove the copper catalyst from an atom transfer radical polymerization (ATRP) [19]. Furthermore, the precipitation of poly(2-ethyl-oxazoline) was performed in glass reactors within a synthetic robot [20]. However, there are only very few publications reporting the automated purification of polymers.

Consequently, the current study focusses on the integration of dialysis for the purification of polymers into automated synthetic robots. Dialysis represents an efficient tool for the purification of polymers, particularly if these polymers can rarely be precipitated [21]. A first approach regarding the automated dialysis of proteins was already described [22]; however, this method is only applicable in water and cannot be implemented into synthetic robots in a simple manner. Furthermore, a high-throughput approach using dialysis in 96-well plate was already studied; however, the volume was limited to a few microliters and the time for an efficient dialysis was more than 200 h [23]. Additionally, this approach is not automated at all, since the preparation of the dialysis was performed by hand. In contrast, the current approach features the benefit of potentially combining synthesis and purification in one automated device. Furthermore, the solvent consumption is reduced and typical organic solvents (not halogenated ones due to the loss of function of the membrane) such as tetrahydrofuran (THF) or acetone can be utilized as well. 

## 2. Materials and Methods

### 2.1. Materials and Methods

All chemicals were used as received from Sigma Aldrich (Merck KGaA, Darmstadt, Germany) (azobisisobutyronitrile, 2-cyano-2-propylbenzodithioat, methyl methacrylate, styrene, anisole, sudan I, crystal violet), VWR (Darmstadt, Germany) (diethyl ether, tetrahydrofuran, chloroform), Thermo Fisher Scientific (Schwerte, Germany) (methanol) and Acros Organics (Thermo Fisher Scientific, Schwerte, Germany) (dimethylformamide), if not otherwise stated. Dimethylformamide (DMF) was dried over a molecular sieve under a nitrogen atmosphere. The liquid monomers that were used, methyl methacrylate and styrene, were destabilized over a short AlOx column (neutral AlOx, obtained from Molecula). The dialysis tubings were purchased from Spectrum Labs^TM^ (Spectra/PorTM, pre-wetted tubing, 3.5 kDa) and were rinsed with the respective solvent before use.

Nuclear magnetic resonance spectra were measured using a Bruker AC 300 (Billerica, MA, USA) (300 MHz) spectrometer at 298 K if not stated differently. The chemical shift is given in parts per million (ppm on the δ scale) related to deuterated solvent.

Size exclusion chromatography measurements (SEC) were performed with the following setup: Shimadzu with CBM-20A (system controller), DGU-14A (degasser), LC-20AD (pump), SIL-20AHT (autosampler), CTO-10AC vp (oven), SPD-20A (UV detector), RID-10A (RI detector), PSS SDV guard/1000 Å/1,000,000 Å (5 μm particle size, supplier: PSS GmbH, separation range: 400–1,000,000 g/mol) chloroform/isopropanol/triethyl-amine [94/2/4] with 1 mL/min at 40 °C, poly(methyl methacrylate) (PMMA) or polystyrene (PS) (standards) (details for the standards are listed in the Supplementary Materials).

Gas chromatography (GC) was performed on a Shimadzu (Duisburg, Germany) with an FID-detector, carrier gas He, and a column (30 m long, 0.25 mm ID, 0.25 µm film thickness) with a stationary phase of 5% diphenyl and 95% dimethyl polysiloxane. As solvent, chloroform was utilized.

All dialysis experiments were performed using a Chemspeed SLT-Accelerator (Füllinsdorf, Switzerland) automated parallel synthesizer (www.chemspeed.com).

### 2.2. Synthesis of the Polymers

#### 2.2.1. Reversible Addition-Fragmentation Chain Transfer (RAFT) Polymerization of Methyl Methacrylate (**P1**–**P2**)

Solutions of the initiator (azobisisobutyronitrile (AIBN)), chain transfer agent (2-cyano-2-propylbenzodithioat), and methyl methacrylate (MMA) in DMF were prepared with a [M]:[CTA]:[I] ratio of 150:1:0.25 in a round bottom flask (M = monomer; CTA = chain-transfer agent; I = initiator). After closing the reaction vessel with a septum, the reaction mixture was degassed by flushing it with nitrogen for 30 min. The solution polymerizations were carried out in a pre-heated oil bath at 70 °C for 17 h. All amounts and volumes of the utilized chemicals are listed in Table 1.

The polymer **P1** was precipitated in diethyl ether and utilized as powder afterwards. **P2** was utilized for dialysis experiments without further purification. The obtained molar masses (number-average *M*_n_ and mass-average *M*_w_) are summarized in Table 2.

**P1**: ^1^H NMR (300 MHz, CDCl_3_, *δ*): 0.78 (s, 3H), 0.95 (s, 2H), 1.68–2.02 (m, 3H) ppm.

#### 2.2.2. Free Radical Polymerization of Styrene (**P3**)

Styrene (15 g, 144.0 mmol) and AIBN (14 mg, 0.08 mmol) were mixed in a 250 mL round bottom flask, and after closing the flask with a septum, the solution was degassed for one hour with nitrogen. The solution polymerizations were carried out in a pre-heated oil bath at 70 °C for 17 h. Polystyrene (1.66 g) was obtained by polymerization and then precipitated from solution with cold methanol (1 L) and after drying in vacuo. 

**P3**: ^1^H NMR (300 MHz, CDCl_3_, *δ*): 1.35 (m, 2H), 1.76 (m, 1H), 6.24–6.68 (m, 2H), 6.80–7.26 (m, 3H) ppm.

### 2.3. Dialysis

#### 2.3.1. Manual Dialysis (Experiments **M1**–**M3**)

The polymer was dissolved in THF (100 mg/mL), poured into a dialysis tubing (3.5 kDa, d = 34 mm) and stored in a beaker filled with 400 mL THF. The surrounding solvent was exchanged every 12 h and a new 400 mL of THF was utilized. In the case of an analysis via GC, 40 µL anisole/mL THF was added to the dialysis solvent. The exact masses and times are listed in Table 3.

#### 2.3.2. Automated Dialysis (Experiments **A1**–**A4**)

The polymer was dissolved in THF (100 mg/mL) and poured into dialysis tubing (3.5 kDa, d = 34 mm) using the four-needle head of the robot. The tubing was fixed to the cap of the dialysis system. The surrounding solvent (250 mL THF) was continuously exchanged by a pump with a volume of 35 mL/hour (modus 1) or changed completely after 3 h (modus 2). In the case of analysis via GC, 40 µL anisole/mL THF was added to the solvent of the experiment. The exact masses and times are listed in Table 3.

#### 2.3.3. Video Automated Dialysis

Crystal violet (5 mg) was dissolved in THF (10 mL). Six milliliters of this solution were transferred into a dialysis tube (34 mm, 3.5 kDa). Additionally, a beaker was filled with 150 mL THF containing Sudan 1 (1 mg/10 mL) and flushed continuously through the dialysis system (5 mL/min). After 10 min, the crystal violet solution was transferred out of the dialysis tube into the product vial. The video was partially speeded up: (6×: 0:08 to 0:17 min and 0:31 to 0:40 min, 5×: 0:55 to 01:15 min; 4×: 1:15 to 1:35 min). The video was interrupted for 10 min at 1:15 min.

## 3. Results and Discussion

**Figure 1:**
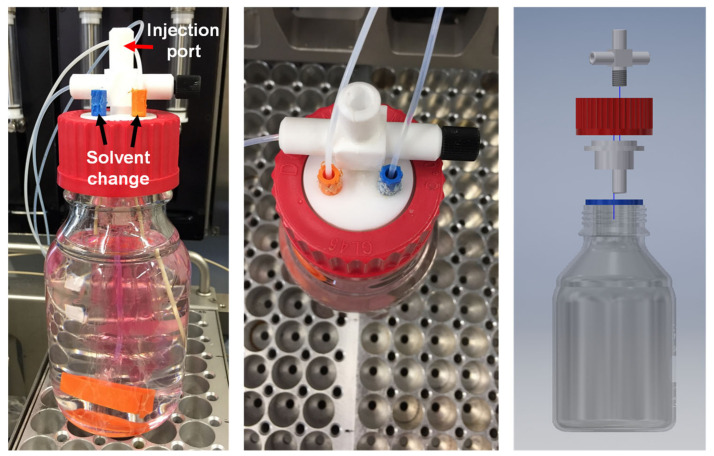
Automated dialysis apparatus installed within the synthesis robot. The 250 mL glass vessel was equipped with a special Teflon cap consisting of an injection port for the four-needle head of the robot as well as possibilities for the solvent exchange (solvent pumping). The dialysis tube is located within the vessel filled with a solution of **P2**.

The automated dialysis setup was constructed using a standard 250 mL bottle and a special Teflon cap enabling the injection via the four-needle head of the robot (Figure 1 and Supplementary Materials). Furthermore, the solvent could be pumped through the system. In the first approach, THF was applied as solvent in order to show the high benefit of using organic solvents and not only water as described in literature for proteins [22]. Furthermore, two modi operandi were developed in order to investigate whether a constant flow (35 mL/h; modus 1) or a complete solvent change (modus 2, change every three hours) is more efficient.

For all experiments, poly(methyl methacrylate) and polystyrene were utilized since these are commonly utilized polymers. Consequently, the working principle and effectiveness can be studied in detail.

**Figure 2:**
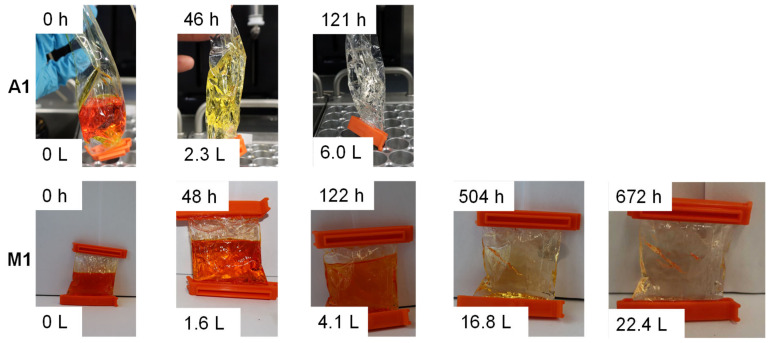
Time-dependent analysis of the dialysis of **P3** and Sudan I mixture (automated dialysis modus 1 top; manual dialysis bottom).

As a first experiment, a dye was added to a polystyrene solution (**P3**). The solution was dialyzed via the manual (**M1**) and the automated procedure (modus 1; **A1**) (Figure 2). The automated dialysis could remove the dye from the polymer solution within 121 h using 6 L of THF. In contrast, the manual dialysis was significantly slower, since after a comparable amount of time and volume, nearly the complete amount of dye was still in the dialysis tube. After 660 h and 22 L the dye could be removed nearly quantitatively from the solution and the dialysis was finished. As a result, the high potential of such an automated approach could be revealed. All pictures can be found in the Supplementary Materials.

**Figure 3:**
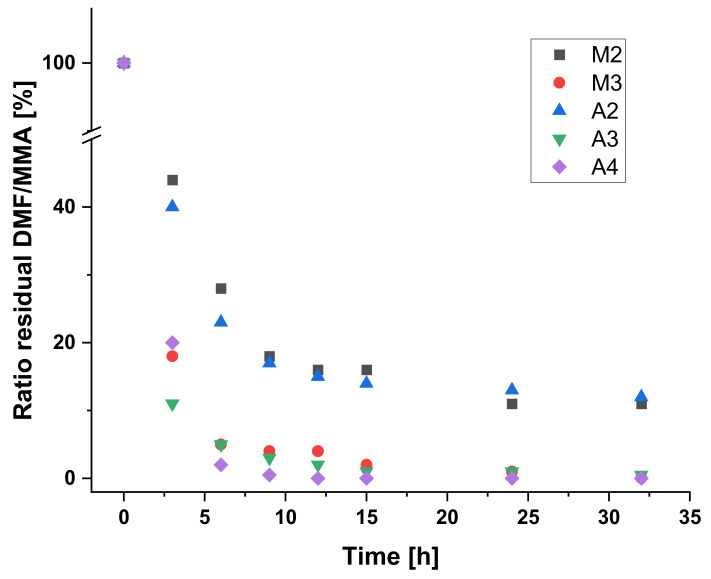
Representation of the decrease in the residual DMF (**M3**, **A3**, **A4**) or MMA (**M2**, **A2**) during dialysis time.

Afterwards, a more polymer-related approach was studied. For this purpose, the polymer **P1** (PMMA) was mixed with a defined amount of methyl methacrylate (MMA) and investigated using a manual (**M2**) and an automated approach (**A2**). The residual MMA was monitored during the dialysis using gas chromatography (GC) (Figure 3) with anisole as standard as well as NMR spectroscopy (see Supplementary Materials). The obtained values are summarized in Table 4. It could be revealed that both dialysis experiments are fairly comparable and that the kinetics are very similar. Therefore, after 32 h, very low residual MMA could be measured in both cases. Thus, the automated dialysis is comparable to the standard manual dialysis for this purification procedure.

**Figure 4:**
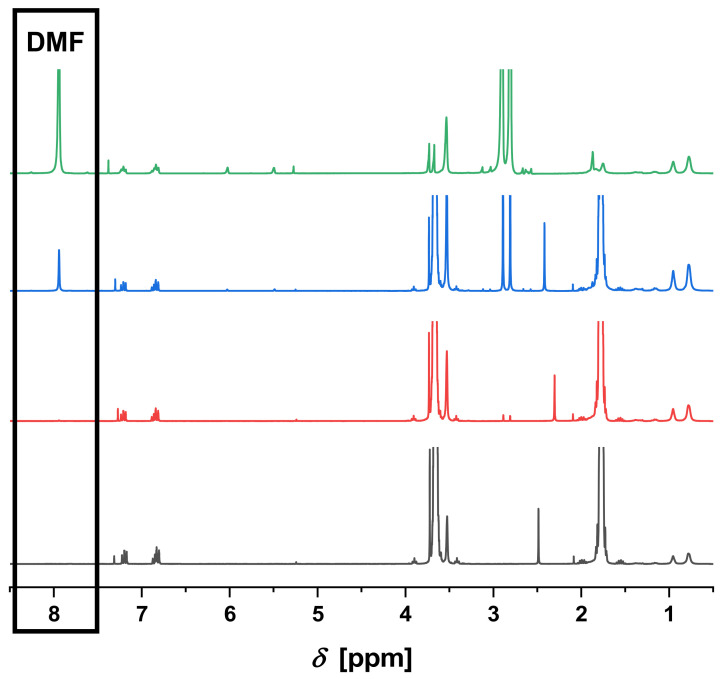
NMR kinetic (CDCl_3_) of the automated dialysis **A4** (modus 2) using the DMF signal at 8.02 ppm (green 0 h, blue 3 h, red 9 h, black 24 h) compared to aromatic anisole signals at 6.75 to 6.90 ppm.

Finally, a reaction mixture was utilized for the purification via dialysis directly after the synthesis without any additional purification steps beforehand. For this purpose, a standard RAFT polymerization of methyl methacrylate was performed (**P2**) and the reaction mixture was directly inserted into a dialysis tube for the manual (**M3**) and the automated (modus 1: **A3**; modus 2: **A4**) dialysis. In these cases, the residual solvent, i.e., DMF was measured using gas chromatography and NMR and was compared to the standard anisole, which was added to the dialysis solvent THF (see Table 4, Figure 3 and Supplementary Materials). The manual and the automated dialysis (modus 1) were also comparable within this experiment. After 32 h, nearly no DMF could be measured anymore. However, the automated dialysis with the application of modus 2 (complete solvent change after 3 h) was significantly faster and was already finished after 9 h (see Figure 4). Therefore, this modus seems to be more suitable for a fast and efficient dialysis. Furthermore, the amount of solvent required for the purification of the polymer is much lower in this case (750 mL after 9 h) compared to modus 1 (9 h: 565 mL; 32 h: 1370 mL) or the manual performance (1200 mL after 32 h). Therefore, the automated dialysis using a complete solvent exchange after 3 h seems to be the most efficient way to purify the polymers.

## 4. Conclusions

Within this study, a new concept for the purification of polymers using an automated dialysis approach was presented. For this purpose, a new dialysis setup was integrated into a synthesis robot enabling a fully automated purification of polymers. Furthermore, two modi operandi were developed using a continuous solvent exchange (modus 1) or a complete solvent exchange every three hours (modus 2). Within this context, the purification kinetics of modus 1 are very similar to a manual dialysis, whereas modus 2 is significantly better compared to the manual or modus 1 dialysis. As a consequence, a lower amount of solvent is required and a fast dialysis can be performed. Consequently, the automated dialysis can be more efficient compared to the manual one in the context of time and required solvent amount. Additionally, the automated procedure requires less purification work on the part of the scientist and, therefore, can provide a significant advantage for the future purification protocols.

Overall, the new setup will enable an automated polymer purification and multi-step reactions of the polymers within the robot, or as a stand-alone system. Furthermore, fast screening and a more automated and digital workflow can be established using the new technology.

## Figures and Tables

**Table 1:** Summary of the utilized amounts and volumes for the Reversible Addition-Fragmentation Chain Transfer (RAFT) polymerizations of methyl methacrylate (MMA).

Polymer	*m*(Monomer) (g)	*V*(DMF) (mL)	*m*(CTA) (mg)	*m*(AIBN) (mg)
**P1**	50	250	736.91	136.68
**P2**	15	75	221.07	41.00

**Table 2:** Summary of the molar masses of the polymers **P1**–**P3** (molar mass was determined using size exclusion chromatography measurements (SEC); standard PMMA for **P1** and **P2**, polystyrene for **P3**; solvent: chloroform/isopropanol/triethyl-amine [94/2/4]).

Polymer	*M*_n_ (g/mol)	*M*_w_ (g/mol)	Ɖ
**P1**	6800	7800	1.14
**P2**	12,600	14,300	1.13
**P3**	42,300	64,000	1.51

**Table 3:** Summary of the dialysis experiments.

Experiment	Modus Operandi	Polymer	Additive	Solvent Change
**M1**	Manual	P3 (500 mg)	Sudan 1 (10.1 mg)	400 mL after 12 h; 55× in total
**M2**	Manual	**P1** (625 mg)	MMA (625 mg)	400 mL after 12 h; 2× in total
**M3**	Manual	**P2** (15 mL solution)	–	400 mL after 12 h; 2× in total
**A1**	Automated (modus 1)	**P3** (500 mg)	Sudan 1 (10.1 mg)	50 mL/h; 121 h
**A2**	Automated (modus 1)	**P1** (625 mg)	MMA (625 mg)	35 mL/h; 32 h
**A3**	Automated (modus 1)	**P2** (15 mL solution)	–	35 mL/h; 32 h
**A4**	Automated (modus 2)	**P2** (15 mL solution)	–	250 mL after 3 h; 5× in total

**Table 4:** Analysis of the efficiency of the manual (**M2**, **M3**) and the automated (**A2** to **A4**) dialysis measured using gas chromatography and NMR spectroscopy. In the case of the reaction solution, the residual solvent (dimethylformamide (DMF)) was measured (**M3**, **A3**, **A4**) (NMR signal at 8.02 ppm compared to signal of anisole at 6.75 to 6.90 ppm), in the case of the other (**M2**, **A2**) the additional methyl methacrylate (NMR signal at 5.5 ppm compared to signal of anisole at 6.75 to 6.90 ppm) was analyzed. The values are given in percentages relative to the sample at the beginning of the experiments.

Time	M2		M3		A2		A3		A4	
**(h)**	GC	NMR	GC	NMR	GC	NMR	GC	NMR	GC	NMR
**0**	100	100	100	100	100	100	100	100	100	100
**3**	44	39	18	16	40	32	11	12	20	18
**6**	28	16	5	5	23	11	5	5	2	2
**9**	18	8	4	3	17	6	3	3	0.5	0
**12**	16	5	4	3	15	4	2	3	0	0
**15**	16	2	2	1	14	2	1	0	0	0
**24**	11	1	1	1	13	1	1	1	0	0
**32**	11	0	0.05	0	12	0	0.5	1	0	0

## Supplementary Materials

The following are available online at https://www.mdpi.com/2073-4360/12/9/2095/s1, Figure S1: NMR kinetic (CDCl3) of the manual dialysis M2 (green 0 h, blue 3 h, red 9 h, black 24 h)., Figure S2: NMR kinetic (CDCl3) of the manual dialysis M3 (green 0 h, blue 3 h, red 9 h, black 24 h), Figure S3: NMR kinetic (CDCl3) of the automated dialysis A2 (modus 1) (green 0 h, blue 3 h, red 9 h, black 24 h), Figure S4: NMR kinetic (CDCl3) of the automated dialysis A3 (modus 1) (green 0 h, blue 3 h, red 9 h, black 24 h), Figure S5: NMR kinetic (CDCl3) of the automated dialysis A4 (modus 2) (green 0 h, blue 3 h, red 9 h, black 24 h), Figure S6: SEC curve of the polymer P1 (blue), P2 (red) and P3 (black) (solvent: chloroform/isopropanol/triethyl-amine [94/2/4]), Table S1: Details of the utilized standards for SEC-calibration. The listed molar masses (Mn) are molar mass at the peak maximum. The supplier of the standards is PSS GmbH, Figure S7: Schematic representation of the technical details of the dialysis apparatus, Figure S8: Automated dialysis equipment without pump (a: picture of the complete device; b: zoom in the injection port; c: zoom in the dialysis tube), Figure S9: Overview of the manual dialysis using Sudan I as dye for the visualization. The solvent was changed every 12 h (400 mL each), Figure S10: Overview of the automated dialysis using Sudan I as dye for the visualization. The solvent was changed continuously (35 mL/h). Video S1.
