# Supplementary material for: Automated Polymer Purification Using Dialysis
Source: Polymers (Basel). 2020 Sep 15;12(9):2095. doi: 10.3390/polym12092095 (PMC7569804; doi:10.3390/polym12092095)
Supplement: Supplementary file 1 [file polymers-12-02095-s001.zip › polymers-922736-supplementary.pdf]

# Automated Polymer Purification Using Dialysis

## Supplementary information

**Timo Schuett [1,2], Julian Kimmig [1,2], Stefan Zechel [1,2] and Ulrich S. Schubert [1,2,*]**

[1] Laboratory of Organic and Macromolecular Chemistry (IOMC), Friedrich Schiller University Jena, Humboldtstr. 10, 07743 Jena, Germany; timo.schuett@uni-jena.de (T.S.), julian.kimmig@uni-jena.de (J.K.), stefan.zechel@uni-jena.de (S.Z.)

[2] Jena Center of Soft Matter (JCSM), Friedrich Schiller University Jena, Philosophenweg 7, 07743 Jena, Germany

* Correspondence: ulrich.schubert@uni-jena.de

**NMR spectra**

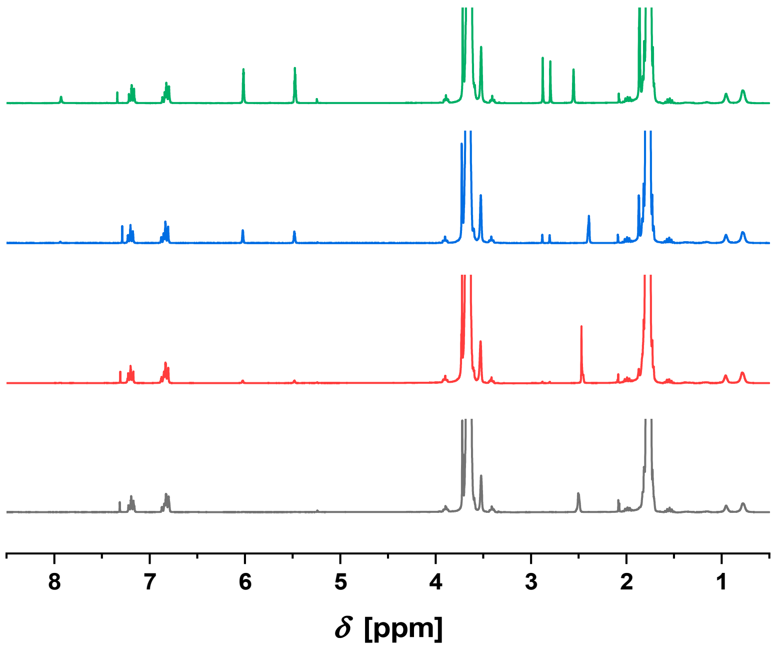


**Figure S1**: NMR kinetic ($CDCl_3$) of the manual dialysis **M2** (green 0 h, blue 3 h, red 9 h, black 24 h).

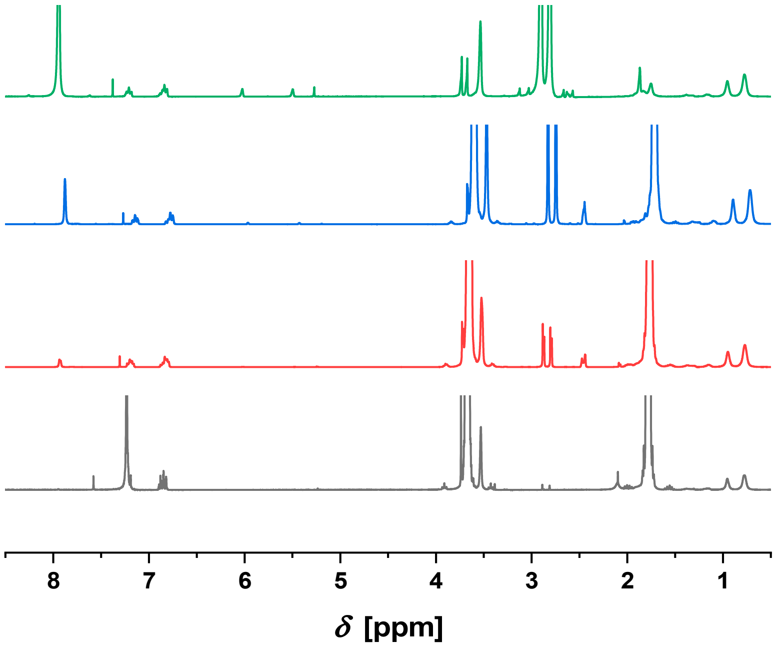


**Figure S2**: NMR kinetic ($CDCl_3$) of the manual dialysis **M3** (green 0 h, blue 3 h, red 9 h, black 24 h).

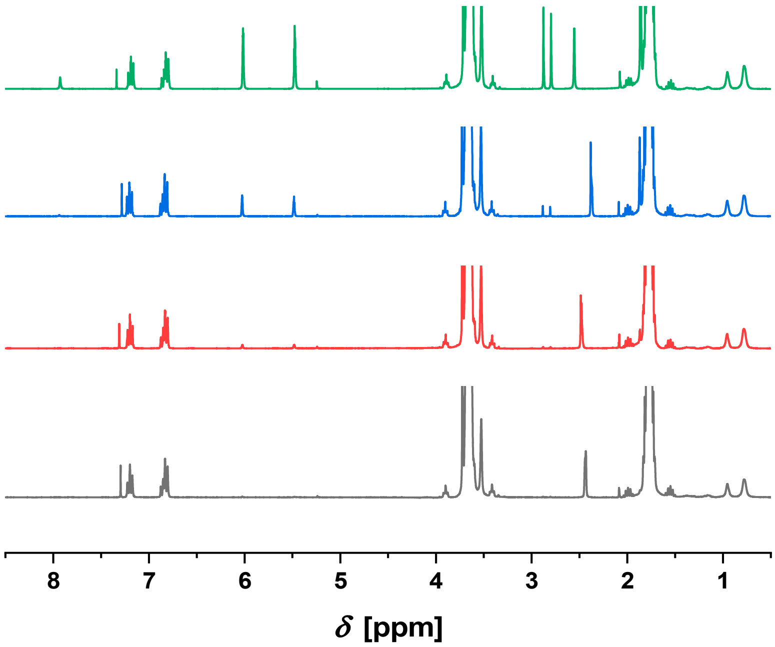


**Figure S3**: NMR kinetic ($CDCl_3$) of the automated dialysis **A2** (modus 1) (green 0 h, blue 3 h, red 9 h, black 24 h).

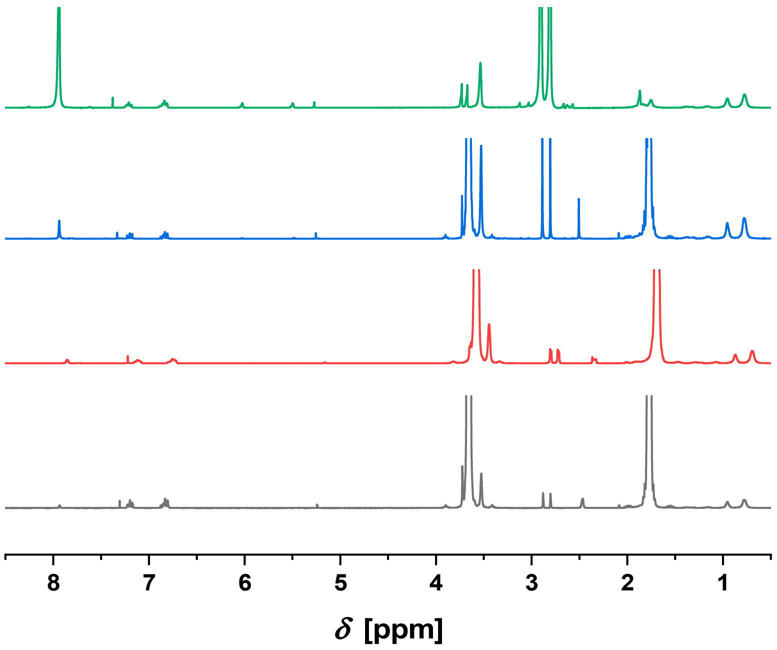


**Figure S4**: NMR kinetic ($CDCl_3$) of the automated dialysis **A3** (modus 1) (green 0 h, blue 3 h, red 9 h, black 24 h).

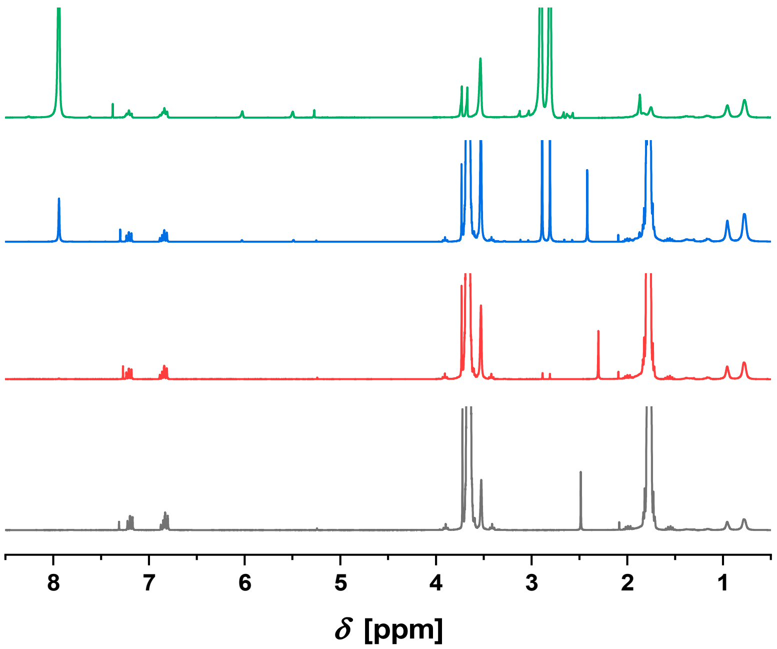


**Figure S5**: NMR kinetic ($CDCl_3$) of the automated dialysis **A4** (modus 2) (green 0 h, blue 3 h, red 9 h, black 24 h).

**SEC curves**

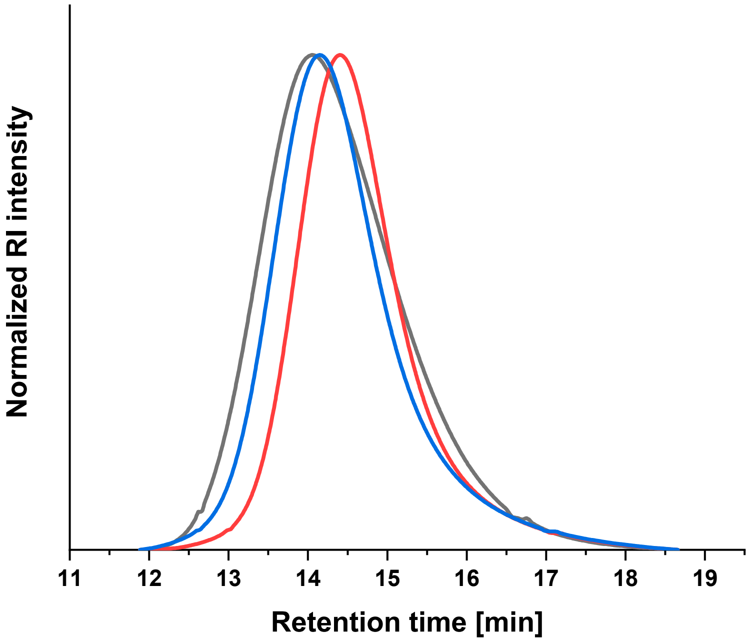


**Figure S6**: SEC curve of the polymer **P1** (blue), **P2** (red) and **P3** (black) (solvent: chloroform/isopropanol/triethyl-amine [94/2/4]).

**Table S1**: Details of the utilized standards for SEC-calibration. The listed molar masses (Mn) are molar mass at the peak maximum. The supplier of the standards is PSS GmbH.

| **Standard** | **Stock solution** | **Molar mass (Mn) of utilized standards** |
|---|---|---|
| Polystyrene | 1 | 1.040.000 |
| | | 128.000 |
| | | 18.100 |
| | | 1.620 |
| | 2 | 659.000 |
| | | 67.500 |
| | | 9.130 |
| | | 685 |
| | 3 | 246.000 |
| | | 32.500 |

| | | |
|---|---|---|
| | | 3.420 |
| | | 374 |
| Poly(methyl methacrylate) | 1 | 981.000 |
| | | 110.000 |
| | | 10.100 |
| | | 1.102 |
| | 2 | 520.000 |
| | | 49.000 |
| | | 4.250 |
| | | 505 |
| | 3 | 217.000 |
| | | 23.300 |
| | | 2.000 |
| | | 102 |

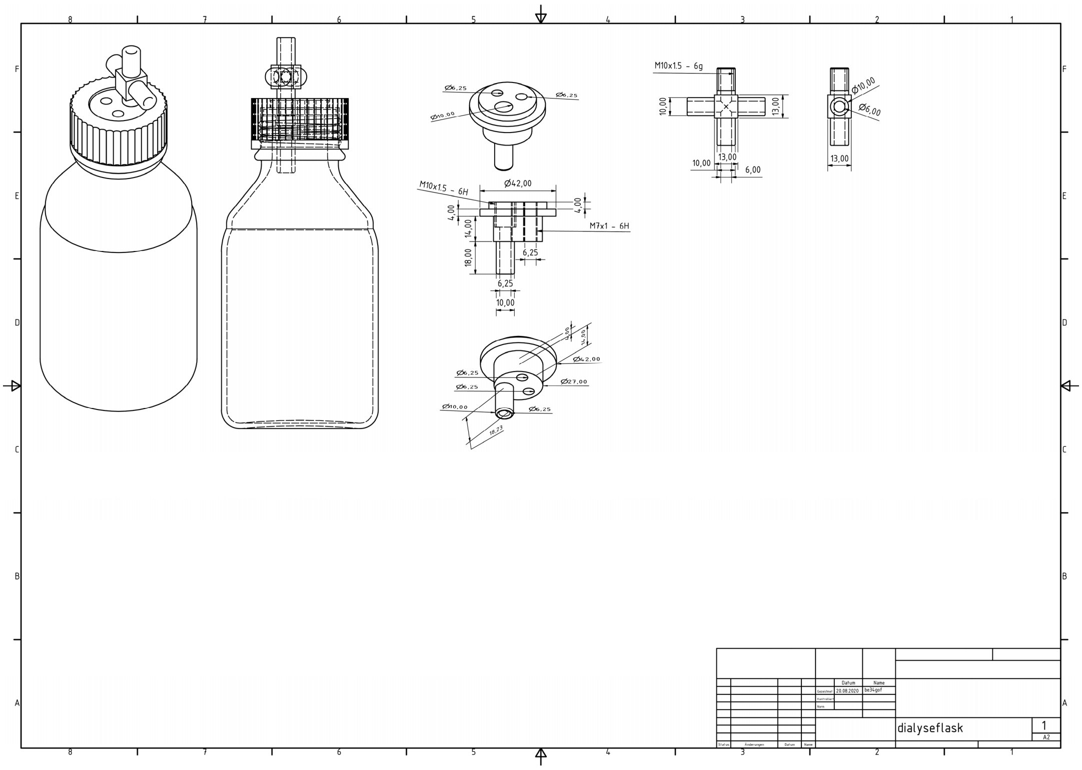


**Figure S7**: Schematic representation of the technical details of the dialysis apparatus.

**Pictures of automated dialysis equipment**

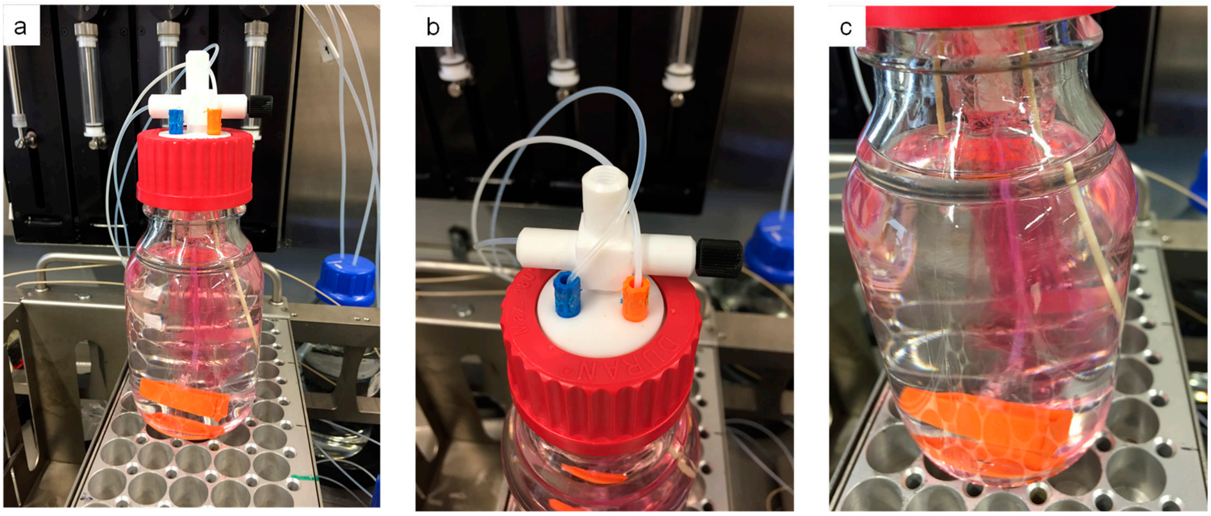


**Figure S8**: Automated dialysis equipment without pump (a: picture of the complete device; b: zoom in the injection port; c: zoom in the dialysis tube).

**Pictures of dialysis M1**

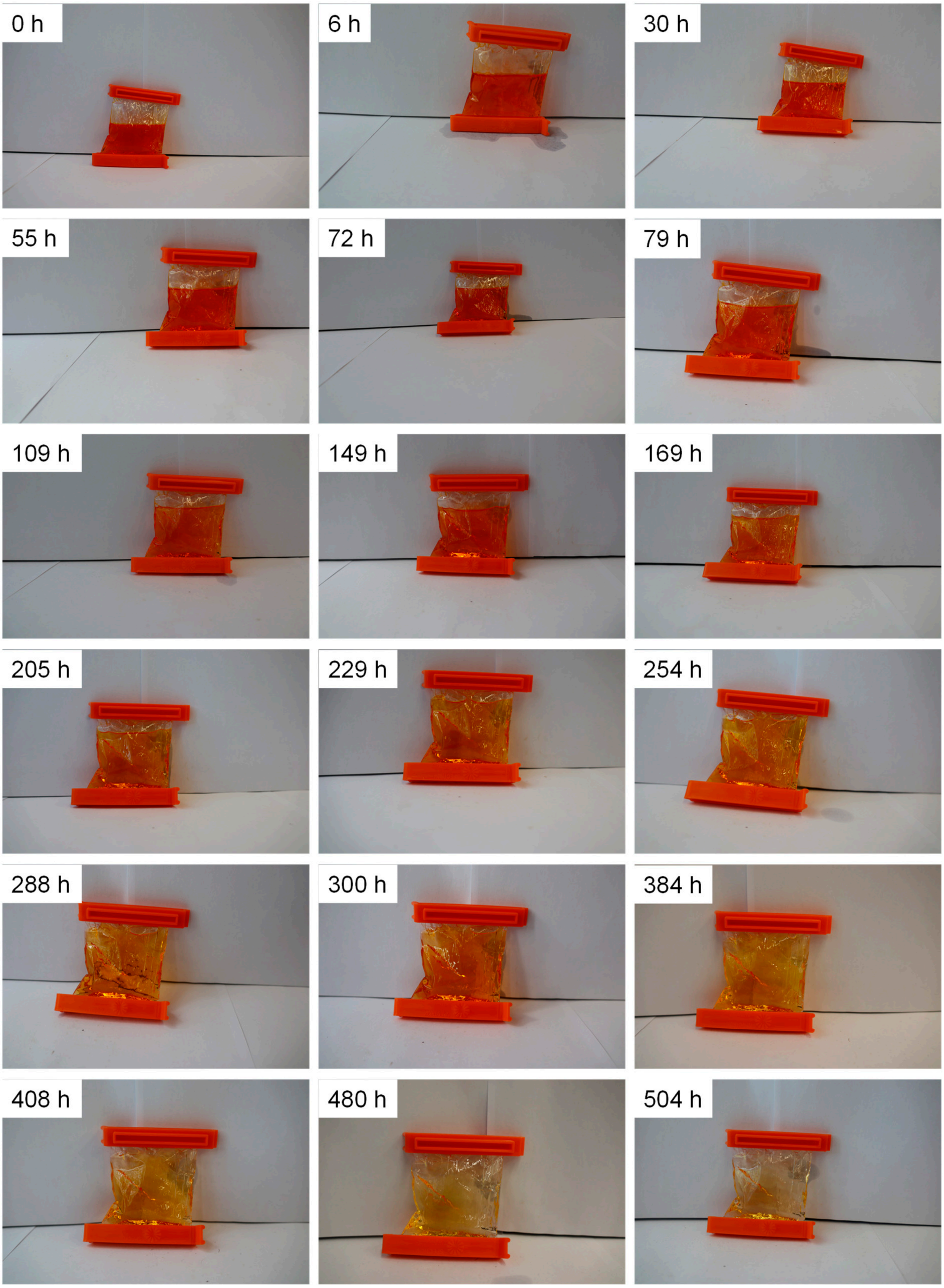


**Figure S9**: Overview of the manual dialysis using Sudan I as dye for the visualization. The solvent was changed every 12 h (400 mL each).

**Pictures of dialysis A1**

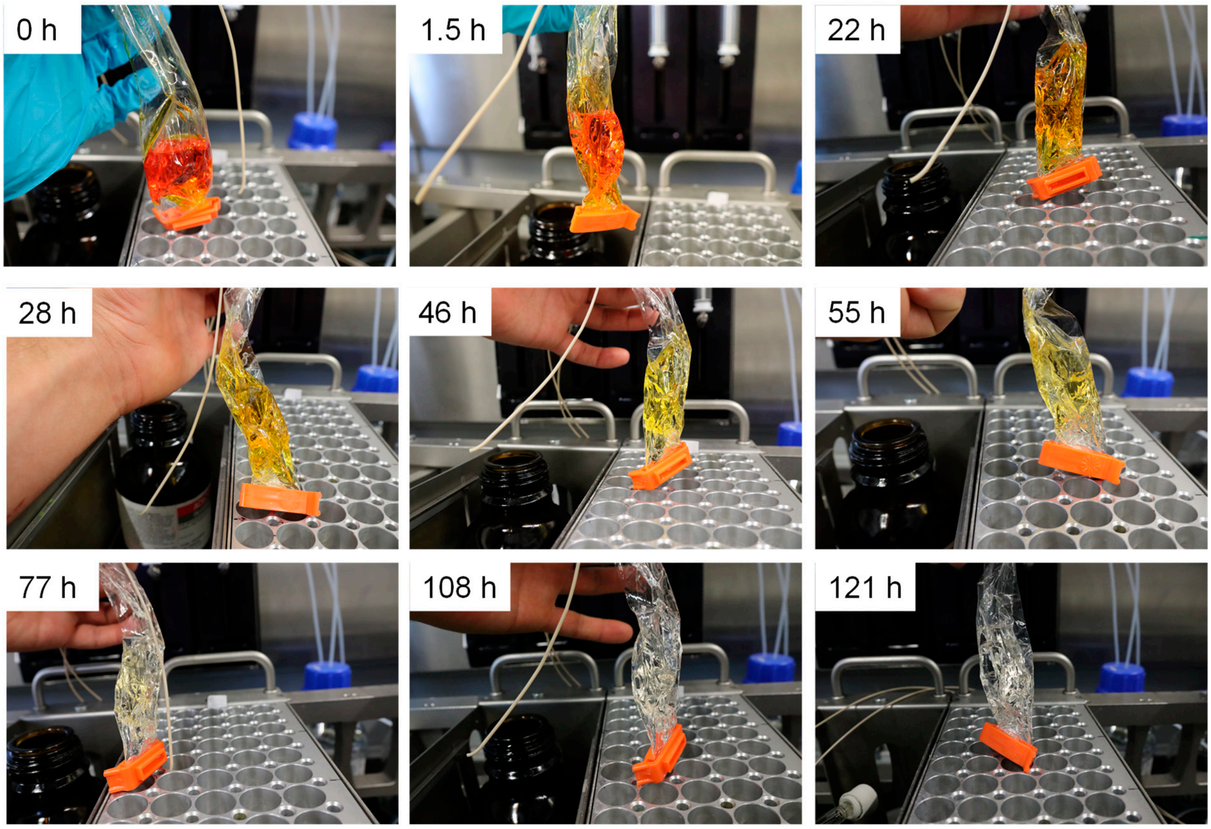


**Figure S10**: Overview of the automated dialysis using Sudan I as dye for the visualization. The solvent was changed continuously (35 mL/h).
